# Avian predation has the strongest impact on vole survival during winter and spring in temperate grasslands

**DOI:** 10.1038/s41598-025-30214-y

**Published:** 2025-12-06

**Authors:** Zbigniew Borowski, Kamil A. Bartoń

**Affiliations:** 1 Forest Research Institute, Sękocin Stary, Braci Leśnej 3, 05-090 Raszyn, Poland; 2 Institute of Nature Conservation, Polish Academy of Sciences, al. A. Mickiewicza 33, 31-120 Kraków, Poland

**Keywords:** Voles, Population dynamics, Winter survival, Birds of prey, Avian predation, Population structure

## Abstract

Predation is a key driver of small rodent population dynamics, yet experimental evidence on the role of avian predators in temperate ecosystems remains limited. We conducted a year-round, spatially replicated field experiment in temperate grassland habitats to test how exclusion of birds of prey affects the survival and abundance of root voles (*Microtus oeconomus*). In each of the three pairs of plots, predators were excluded from one plot by overhead netting. Vole survival and population size were assessed using capture–mark–recapture method, considering the effects of sex and body mass. Avian predation reduced vole survival by up to 22%, with the strongest effect in winter and spring. Predator exclusion led to short-term increase in vole numbers during spring, but did not produce sustained differences in abundance. The effect of avian predation was not selective regarding sex, but heavier voles showed reduced overwinter survival. Effect of predation varied among sites, with the strongest impact observed at the location nearest to a forest edge. Our results demonstrate that avian predators can substantially contribute to vole mortality in temperate open habitats, even under snow cover, and highlight how seasonal predation may be altered by climate-driven changes in snow conditions and vegetation phenology.

**Supplementary Information:**

The online version contains supplementary material available at 10.1038/s41598-025-30214-y.

## Introduction

Predation can exert profound impact on the dynamics and demographic structure of prey populations, but this impact can vary considerably depending on characteristics of both the predators and prey, as well as on environmental conditions^1^. Small rodents, including voles and lemmings, are particularly interesting example in this context, as their high abundance and rapid reproduction make them staple prey to a broad range of predators of widely varying sizes and hunting strategies^2–4^. Predation can both limit and regulate rodent populations, and can reach high levels, leading to population declines. For example, a study in Sweden reported that predation delayed and reduced annual recovery of the rodent population^5^. The intensity of predation pressure on small rodents depends on several interrelated factors, including the phase of the rodent population cycle, habitat characteristics, and the structure of the predator community^4^. Although predation on small mammals has been extensively studied in boreal and arctic ecosystems, corresponding research from temperate regions remains limited^6^.

Predator communities preying on small rodents are typically composed of both specialist and generalist predators^2^. Among specialists, small mustelids often are key predators, whose numerical response tracks rodent densities with a time lag, inducing delayed density dependence that can drive cyclic population fluctuations^7^. In contrast, generalists and nomadic predators can stabilise prey dynamics both in time and in space, by rapidly switching to an alternative prey or following local changes in prey density^8,9^. Birds of prey are a major group of such predators and constitute an essential component of predation pressure on small rodents^9,10^. The impact of avian predators on rodent populations varies geographically and is often intertwined with the impact of mammalian predators^7,11,12^.

Within avian predators, responses to changes in prey availability differ between migratory and resident species. Migratory raptors track broad, seasonal changes in vole abundance, whereas non-migratory species make immediate behavioural adjustments to local prey fluctuations. Residents adjust by shifting home ranges, altering habitat selection, or switching to alternative prey. Migratory birds, by contrast, are constrained by their migration schedules and may be less able to respond to local fluctuations^13,14^, showing a lagged numerical response to vole abundance^12,15^. Consequently, the intensity of avian predation varies depending on the phase of the vole population cycle. Predation rates by birds can be significantly higher during the peak and decline phases^12,16^, in some cases reaching up to five times the levels observed in other phases of the vole cycle^9^.

In seasonal environments, avian predation pressure on small rodents can fluctuate markedly over the year, reflecting changes in prey vulnerability and availability, and predator behaviour(e.g.^17^). Most studies of small rodent populations have been conducted in northern latitudes(e.g.^12,18^), whereas the role of seasonal predation can vary geographically(e.g.^19^), and in temperate environments is thought to have less impact on vole dynamics^19–21^. Winter is a particularly critical period, when voles face limited food resources, high energy expenditures, while mortality is not balanced with reproduction^18,22^. Predation, including avian predation, has been shown to contribute to winter declines of voles^23,24^. Nevertheless, relatively little attention has been given to the specific role of winter avian predation, partly because few raptor species remain through winter and snow cover limits hunting efficiency in boreal and arctic ecosystems(^18,25,26^). Most experimental work has therefore focused on vole survival during winter without distinguishing between avian and mammalian predation^18,25–27^.

Avian predators are often selective with respect to prey species or demographic groups, and this selectivity often varies seasonally. For example, kestrels and owls preferentially prey on the heaviest voles in winter, while in summer subadults and males are taken more frequently^16,28^. In contrast, mammalian predators may show less pronounced or even opposite biases^15,29^. Such patterns of selective predation can have substantial consequences for rodent population structure and dynamics throughout the year^3,16,23^.

Predation by birds is also influenced by habitat structure, including openness and the availability of perches^16,30^. In temperate open habitats, predation pressure may vary seasonally depending on plant phenology and climate. Structural elements such as tussocks and snow cover can reduce the efficiency of avian predation, as evidenced by seasonal changes in the diet composition of avian predators^31^.

Experimental approaches that manipulate predation pressure—either by excluding or maintaining predators—are essential for assessing its impact on prey populations^32,33^. To date, however, only a few experiments have excluded avian predation, and these were primarily limited to boreal ecosystems or enclosed populations^23,24,33,34^. In the boreal zone, reducing bird predation has been shown to increase field vole (*Microtus agrestis*) numbers and to reverse the typical summer decline in abundance^23^. Similar results were obtained in aviary experiments with enclosed vole and lemming populations, where excluding both avian and mammalian predators altered abundance dynamics and reversed decline phases^24,27,34^.

In this study, we assess the impact of avian predation on free-living root voles (*Microtus oeconomus*) in temperate grassland habitats. We hypothesised that predation by birds of prey substantially reduces vole survival. Specifically, we predicted that reducing avian predation would (i) increase vole survival during spring, (ii) result in higher vole densities at the onset of the breeding season of voles, and (iii) reveal demographic selectivity with respect to sex and body mass, with males and smaller individuals experiencing higher predation risk due to their greater activity levels and use of open microhabitats^28,35,36^. To test these predictions, we conducted a spatially replicated field experiment in which avian predation was excluded and vole populations were monitored throughout the year.

## Results

**Fig. 1:**
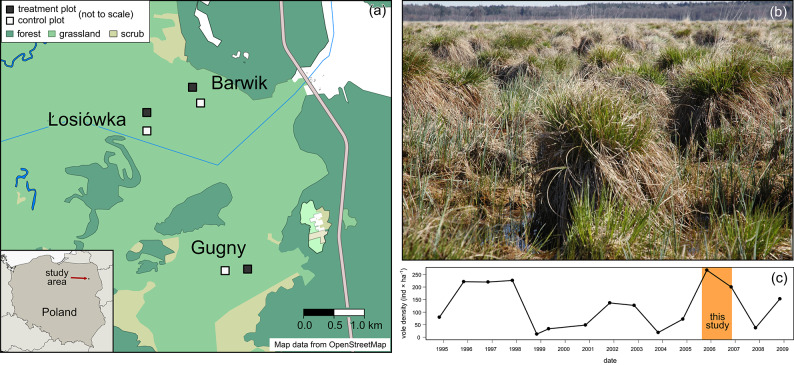
(**a**) Map of the study area, with locations of the experimental and control plots (**b**); view of the study site (photo by Z. Borowski); (**c**) long-term population dynamics of the root vole *Microtus oeconomus* based on spring and autumn densities in Biebrza National Park in 1994–2008^42^. The shaded area indicates the period of the avian exclusion experiment presented in this work. The map was created with data from OpenStreetMap (https://www.openstreetmap.org/copyright) using R environment version 4.4.1 (https://www.R-project.org) with packages ‘osmdata’ (https://docs.ropensci.org/osmdata/) and ‘sf’ (https://r-spatial.github.io/sf/).

In total, we recorded 3984 captures of 1731 individuals, of which 59.2% (n = 1025) were recaptured at least once (see Table S1 in the ESM). On average, 57.4 (± 15.1 s.d.) individuals were caught in each session and plot (Fig. 1), with numbers ranging from 26 to 94 (see Table S1 in the ESM). Individuals were captured on average during 1.6 (± 1.0 s.d.) sessions, or on 2.3 (± 2.0 s.d.) occasions (days). Of all captured individuals, 63% (n = 1093) were recorded during a single session, of which 831—only on one occasion (i.e. one day). The average period over which voles were seen (from first to last capture) was 48.7 (± 81.9 s.d.) days (with a maximum of 455 days). Nine individuals were recorded for the entire duration of the study (i.e. those from the first cohort who were also recaptured during the last trapping session), of which three in every session (see Table S3 in the ESM).

### Model selection

The four best-ranked capture-mark-recapture models contributed a total of 99% of the model weight (*ω*) (Table 1). For survival, *S,* in addition to the variables a priori included in all models (i.e. location, time and body mass), the best-ranked models incorporated the effect of treatment in interaction with either location, time, or body mass. For capture probability, *p*, besides the effect of location interacting with time which was fixed to all models, the highest-ranked models included time, body mass interacting with time, and location interacting with time within session. The time effect in these models was included either in a categorical form or as 3- or 4-degree spline function. Cohort and sex were only included in lower-ranked models. Therefore, model selection suggests that the experimental treatment was overall an important factor for survival, and both the survival and the effect of body mass on survival varied through time. We used AIC_*c*_ for the above model ranking, since the bootstrap goodness-of-fit test showed no lack of fit of the models to our data (*p* = 0.6, *ĉ* = 1.02), indicating no need for quasi-likelihood adjustment. Six models had effectively non-zero weights (taken to be *ω* > 0.001), and all the estimates reported below are averaged over these models.

**Table 1:** Model selection table.

Survival model (S)									
Location	Treatment	Time	Body mass	Cohort	Location × treatment	Location × time	Treatment × time	Treatment × body mass	Time × body mass
+	+	[4]	+			r	[3]		[3]
+	+	+	+		+	r		+	[3]
+		+	+		+	r			[3]
+		[4]	+	+	+	r			[4]

Capture probability model (*p*)					k	ΔAIC_*c*_	Model weight ω_AIC*c*_
Location	Sex	Location × time	Location × session day	Location × time × session day			
+		[4]	+	[3]	58	0.0	0.46
+		[4]	+	[4]	63	0.9	0.30
+	+	[4]	+	[4]	63	2.8	0.11
+		[4]	+	[4]	67	3.8	0.07

Rows are showing terms included in each capture-mark-recapture model, with number of parameters (k), relative AIC_c_ value, and ‘Akaike weights’ ωAIC_c_. Only highest-ranked models with cumulative ‘Akaike weights’ ω_AICc_ ≤ 0.99 are included. Two components are given: model for capture probability, *p* and for survival, *S*; the remaining component models were identical. The ‘ + ’ denotes the presence of a term in a model. For model terms involving time, ‘[3]’ and ’[4]’ refer to the form of time effect included (as a smooth spline of 3rd or 4th order respectively), ‘ + ’ and ‘r’ denote time as a categorical variable, with ‘r’ indicating that the last two sessions have been combined. For consistency, ‘time’ in both components (*S* and *p*) refers to a session (i.e. primary sampling), and ‘session day’ refers to the ordinal day within each session (secondary sampling), which is different from the convention used in the program ‘Mark’, where ‘time’ in the capture probability model refers to secondary sampling.

### Survival

**Fig. 2:**
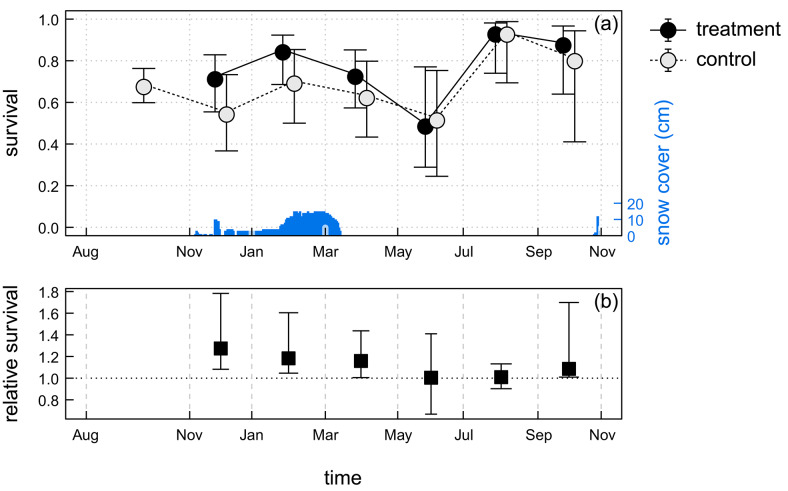
(**a**) Average survival of root voles during the study, in treatment (no bird predation) and control plots, for intervals between sessions and scaled to a one-month period; and (**b**) relative survival for experimental treatment (ratio of survival in treatment and control plots). Points show expected values, whiskers represent 95% confidence intervals. Estimates are averaged over a set of highest-ranked models (see Table 1). Consecutive points from the same plot are connected by a line, points from the same session are shifted apart along the horizontal axis. Daily depth of snow cover is shown at the bottom axis with blue bars.

For about the first half of the study period (November to May), mean survival in the treatment plots was higher than in the respective control plots (Fig. 2; Figure S1 in the ESM). Relative survival, defined as a ratio of survival in treatment plots to that in control plots, was highest during winter, from November to March, with survival in control plots lower by 15%–22% than in treatment plots (*p* ≤ 0.003). Overall, the temporal pattern of survival was similar between plots, but differed slightly in the timing and magnitude of peaks and troughs. Estimated survival increased between January and March (until May at Barwik site), followed by a decrease between May and July, and then another increase, which was highest at Barwik. A period of high survival coincided with a low average body mass, and conversely, a decrease in survival occurred when captured individuals were heavier (Figure S2 in the ESM).

### Body mass

The effect of the experimental treatment on body mass was intermittent and varied between sites (Figure S2 in the ESM). Overall, the proportion of heavier individuals (above ca. 30 g) decreased in winter, followed by an increase that started in spring (Table S5 in the ESM). After this increase, between May and July, the higher proportion of heavier individuals persisted for a shorter period in the treated plots than in the control plots. However, this effect was only significant in Barwik and to a lesser extent in Losiowka.

**Fig. 3:**
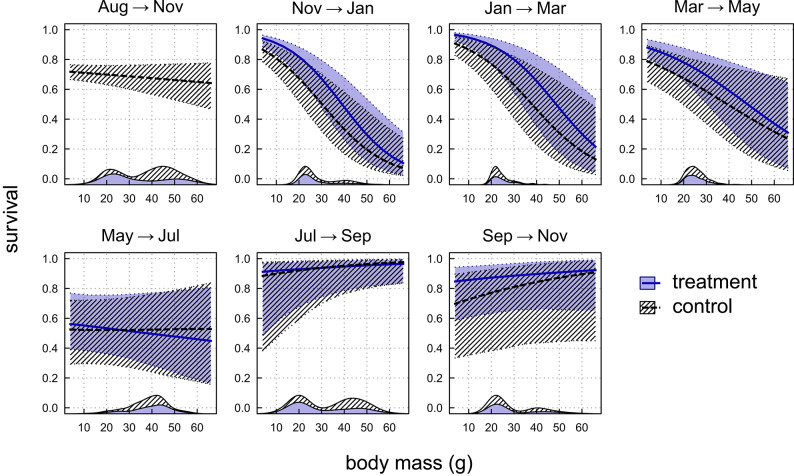
Relationship between individual survival and body mass of root voles for between-sessions periods. Thick lines show expected values, shaded areas cover 95% confidence intervals. Estimates are based on the parameters of highest-ranked models and model-averaged (see Table 1). No treatment was applied in the first session. The body mass density distribution of voles (at the beginning of a given period) is shown at the bottom of each panel at x-axis (for control and treatment plots, stacked).

The relationship between body mass and survival changed during the study (Fig. 3). In the first period, from November to May, survival decreased with body mass, thus the largest individuals faced highest mortality. The average body mass of individuals captured over that period was 24.9 ± 4.6 g (range 16–44 g). In subsequent period (i.e. from May onwards) the models showed no effect of body mass on survival. This diminishing relationship between body mass and survival coincided with an increase in the proportion of larger individuals (in May, see Fig. 3; Figure S2 in the ESM), but as the share of larger individuals decreased again, the relationship remained flat.

### Population size

**Fig. 4:**
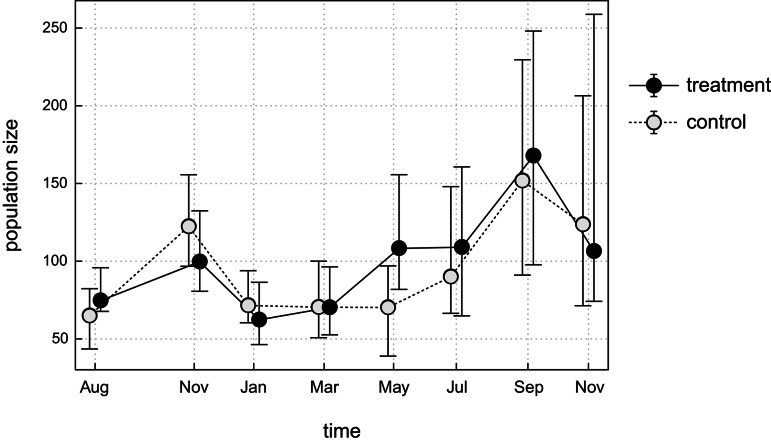
Population size of root vole during the study in treatment and control plots. Points show expected values, whiskers represent 95% confidence intervals. Estimates are averaged over a set of highest-ranked models (see Table 1). For readability, consecutive points from the same plot are connected by a line, and points from the same session are slightly shifted apart along the horizontal axis.

The estimated population size varied substantially during the study, as well as between locations (see Fig. 4; Figure S3 in the ESM). However, there were common patterns, which included a peak before the onset of winter, a winter decline, and a subsequent increase that continued through the end of the study. Initial population sizes, prior to netting, differed within each pair of plots, with the control populations being less numerous in each location (on average in 74.5 (67–96) individuals in treatment, and 64.8 (43–82) in control plots, the mean percentage difference was 14%, see Table S6 in the ESM). Relatively largest difference was between plots at Losiowka (25 individuals or 43%). The differences in the estimated population size in the treatment plots relative to the control plots shifted to negative after the start of the experiment, in November, and reached similar level in all plots (on average − 20.8 individuals, see Figure S4 in the ESM). Population size in all treatment plots again exceeded that in the control plots only in May, reaching its maximum (48.0 individuals on average). In Barwik, this started already in March and remained so until the end of the study. In contrast, on the other plots, the absolute differences in estimated abundance decreased and remained at low levels (− 1.6 individuals on average). These fluctuations suggest that the initial differences in population sizes between the treatment and control plot pairs were not related to habitat quality and are unlikely to systematically affect the results.

The observed increase in population size that started in spring was stronger in plots where avian predation was excluded (Fig. 4). It was particularly apparent between March and May, when population size in all treatment plots was larger that in the corresponding control plots (see Figures S3 and S5 in the ESM). At Barwik, the faster population increase in the treatment plot had a lasting effect, with the population size remaining consistently larger than in the control plot until the study was concluded (Figure S4 in the ESM). In the other plots, exclusion of bird predation did not cause clear long term effect in terms of population size.

## Discussion

By experimentally preventing birds of prey from accessing free-living voles throughout the year, we were able to analyse the role of avian predation both during and outside the vole breeding season, including in winter. We demonstrated that avian predation can substantially reduce vole survival—by up to 22% at its maximum in winter and spring. However, predator exclusion had no prolonged effect on vole abundance, suggesting that avian predation alone may not be able to determine vole population dynamics in temperate ecosystems. Avian predation was not selective for the sex of voles, and while survival decreased with body mass during winter, this relationship was not altered by predator exclusion.

In line with our first prediction, we found that restricting birds of prey increased vole survival in spring, but it was also surprisingly high in winter. This resulted in a short-term increase in abundance during March–May, partly supporting our second prediction. Based on the average survival rates, we can estimate that birds of prey accounted for 44% (19–72%) of mortality at its peak in late winter (January–March). However, this figure should be interpreted with caution because of the high uncertainty, related to relatively high survival on these occasions. Moreover, the effect of predation removal may be underestimated, because voles established at the edge of the plots had parts of their home ranges outside the netted area and thus remained exposed to predators. This finding is in agreement with an experimental study in Finland, where avian predators accounted for about 23% of vole mortality in spring^37^, and in Norway, where they contributed 10–30% of predation on reproductive females^9^.

Unlike in northern cyclic populations of voles, where summer declines in density are common^38^, we did not observe such crashes in our study populations. These summer declines are thought to be driven by non-random prey selection by small mustelids^6,7^, avian predators^9^, or synergic impacts of all predators^12^. The observed reduced survival in May–July reflected cohort turnover, as voles born in the autumn of the previous year, maturing and gaining weight rapidly in spring, experienced high mortality (cf. “class 3” in Figure S5 in the ESM). Only about 10% of these voles were recaptured later, indicating that mortality rather than emigration explained their disappearance from the population. This is substantiated by our modelling results, showing that the negative relationship between body mass and survival persisted only until summer. Thus, low survival was not analogous to the summer declines linked to predator-driven mortality in northern populations, but rather reflected demographic processes.

The observed spring increase in vole numbers in the reduction of avian predation (March to May) coincided with both the arrival and breeding season of bird species that prey on voles and the onset of the root vole breeding season^39^. In early spring, meadows provide the fewest shelters, making voles more vulnerable to attacks by birds of prey. Additionally, at the beginning of the breeding season, the open wet meadow in our study area is typically flooded and vegetation is sparser, making it easier for predators to locate rodents. When vegetation becomes dense around July, it acts as a cover for voles from bird predators. This finding aligns with the results of earlier studies showing that the impact of birds of prey on the vole population is greatest in spring^11,37,40^.

Long-term observational and experimental studies show that avian predation on voles is density-dependent, intensifying during periods of high vole abundance^10,15,41,42^. Studies suggest that avian predators such as kestrels, owls, and harriers often aggregate in areas of high vole density during the increase and peak of rodent population, thereby intensifying predation pressure(e.g.^42^). In contrast, predation rates drop during the low phase, when predators switch to alternative prey or disperse to other areas^12,29^. Some evidence indicates that avian predation can be particularly strong during the decline phase due to a lagged response, thus contributing to population crashes(e.g.^9,23,43^). The relatively limited effect of avian predation observed in this study may therefore be related to the timing of the vole population cycle. As our study was conducted during the peak phase^39^, it is possible that avian predation was at its maximum during the study period. However, if birds of prey respond with a time lag, stronger and more prolonged effects would be expected during the decline phase.

We found that the highest proportion of mortality attributable to avian predators occurred in winter, underscoring the importance of this season for vole demography. Winter conditions may be a key determinant of vole density at the onset of the breeding season and thereby shape both seasonal and multiannual dynamics of vole populations^18,22,24^. Previous work has shown that high winter predation can outweigh summer reproduction and dampen cyclicity^44^. In our study, permanent snow cover persisted from December to March, which may have offered some protection against bird predation^45^. However, owls may use acoustic signals to hunt small mammals under the snow^46^. Moreover, the snow layer was relatively thin (≤ 15 cm), which could facilitate prey being located by owls. Indeed, tawny owls and long-eared owls, both present in our study area^39^, are known to hunt successfully under snow. Analyses of owl diets in similar habitats confirm that arvicoline rodents dominate their winter prey(e.g.^31,47^). These observations help explain why avian predation remained an important source of mortality even in snowy conditions.

The effect of avian predation varied across the locations, which emphasises the importance of local context. The strongest impact was observed at Barwik, located closest to the forest edge (300 m), whereas the other two sites were around 900 m away from the forest. The structure and density of herbaceous vegetation, as well as the distance from the forest edge, can significantly impact bird predation^48,49^. Forest proximity likely intensified predation by providing habitat for woodland raptors such as tawny owls, which often hunt voles outside the forest, as well as suitable perching sites^16,30,50^, and shelter from adverse weather. These fine-scale differences in habitat and landscape structure may therefore play an important role in shaping avian predation.

Contrary to our third prediction, we did not detect sex-related differences in the susceptibility of voles to bird predation. Earlier studies suggested that males are more vulnerable to bird predation due to greater activity^51–53^. These differences are most likely to manifest during the reproductive season. However, we considered only the average effect of predation on each sex, i.e. without seasonal variation, and voles were not sexually active for most of the study period. Reduced sex-related behavioural differences outside the breeding season may have obscured the overall selective predation effect. Similarly, a study of pygmy owl predation^28^ also found no selectivity with regard to prey sex outside the reproductive period. On the other hand, we observed a clear relationship between body mass and overwinter survival, whereby heavier individuals experienced higher mortality. This may be due to the higher energetic requirements of larger voles during seasonal food shortages and hence increased risk of starvation^18,22,54,55^, as well as to age-related mortality, since older voles are generally heavier^22,39^. Predator exclusion did not affect this pattern in our analysis, but an examination of capture histories suggests that the heaviest voles showed the largest differences in survival between control and treatment plots (cf. “class 2” in Figure S5 and Table S2 in the ESM for details). This observation is supported by previous reports that owls and kestrels preferentially prey on heavier voles in winter^12,16^. This is likely due to large individuals being more visible or active above the snow, or to increased risk-taking behaviour in individuals with higher energetic requirements^16,56^.

## Conclusions

Our findings demonstrate that avian predators can substantially reduce vole survival in temperate grasslands, particularly during winter and spring, a critical period for small mammal populations in seasonal climates. Although exclusion of birds did not produce sustained increases in vole abundance, predation accounted for a considerable proportion of mortality and shaped short-term dynamics. These effects varied with local habitat structure and individual traits, highlighting the importance of spatial heterogeneity and demographic context on predation pressure. Crucially, we show that winter mortality can be attributed in part to avian predation even under snow cover, a finding with direct relevance for understanding how changing winter conditions will affect rodent populations. As climate change continues to alter snow cover and vegetation phenology, the seasonal timing and intensity of bird predation may shift, with consequences for both vole dynamics and predator communities. Finally, our observations underscore the role of individual seasonal variation in body mass, which may contribute to shaping population dynamics through differential mortality, pointing to an underexplored but interesting area for future research.

## Material and methods

### Study area

The study was conducted in the Lower Basin of the Biebrza National Park, NE Poland (53°36′18″N, 22°55′36″E). The study area is located in a homogeneous sedge wetland with the vegetation dominated by plants of the Cyperaceae family. The main plant species in the Park is the fibrous tussock sedge *Carex appropinquata*, which covers 85% of the area and forms hummock–hollow structures with tufts up to 1.5 m high^57^ (Fig. 1). In places, the sedge meadow is interspersed with shrubbery, including willows, birches and alders, indicative of an early stage of secondary succession. The wetland has a seasonal water regime with the highest level in spring when flooding is frequent. However, no spring flooding was recorded during the study period. The climate of the area combines continental and subboreal features, with long winters (> 100 days), a short and early spring, and a short growing season (77–85 days). The coldest month is February and the warmest is July, and the total annual precipitation is 550 mm. The winter (2005/2006) was characterised by permanent snow cover from December to the end of March. The meteorological data on the duration and thickness of the snow cover were obtained from the Biebrza National Park meteorological station.

The community of diurnal birds of prey observed near the trapping grids included the common buzzard (*Buteo buteo*), rough-legged buzzard (*B. lagopus*), Western marsh harrier (*Circus aeruginosus*), hen harrier (*C. cyaneus*), and Montagu’s harrier (*C.* *pygargus*), as well as the lesser spotted eagle (*Clanga pomarina*)^39^. The area is also home to a resident year-round population of tawny owls (*Strix aluco*) and long-eared owls (*Asio otus*), as well as seasonally migrating short-eared owls (*Asio flammeus*). The main mammalian predators of the voles were the red fox (*Vulpes vulpes*) and small mustelids (the least weasel *Mustela nivalis,* and the stoat *M.* *erminea*)^39^.

Rodents constitute the majority of small mammals in this area, and root voles are the dominant rodent species in this habitat, accounting for more than 90% of the small mammal community^39,58^. Apart from the root vole, a small number of individuals of other rodent species were also caught, including the harvest mouse (*Micromys minutus*) and yellow-necked mouse (*Apodemus flavicollis*). Two species of insectivorous mammals were also captured: the common shrew (*Sorex araneus*) and the pygmy shrew (*S. minutus*). The natural root vole population studied in this work is characterised by multi-annual, four-year abundance cycles^39,57^. The study began in 2005 during the vole population peak phase and was continued in 2006 during the decreasing phase of the vole population cycle (Fig. 1).

### Experimental setup

We carried out an avian exclusion experiment from November 2005 to November 2006, i.e. spanning one winter. Three pairs of trapping grids (50 × 50 m, 1–3 km apart, see Fig. 1) were established in August 2005 in locations chosen to minimise variation in vegetation and topography among them. The three locations of the trapping grids: Barwik, Losiowka and Gugny were chosen based on visual assessment of habitat similarity, considering factors such as vegetation type, height, structure, and the absence of tall trees and shrubs. Each pair contained one experimental and one control plot, spaced approximately 300 m apart to reduce the probability of movement of voles between plots. The grids were open and voles were able to roam freely. All grids were equipped with 36 permanent trap stations in a 6 × 6 grid with 10 m spacing. Each trap station consisted of one live trap. The population of voles was surveyed by live trapping, with five-day trapping sessions taking place at two-month intervals throughout the experiment. Wooden live traps with metal doors were baited with oat seeds and checked twice a day, in the morning at 8:00 and in the evening at 19:00. The experiment comprised seven trapping sessions, starting from November 2005. Before the start of the experiment, we conducted one trapping session in August 2005 to compare the control and experimental grids in each location. During the winter and early spring sessions (January, March), when ambient temperatures were lowest, traps were opened only during the day (from 8:00 to 19:00) to reduce trap mortality. Likewise, during the summer session (July), when ambient temperatures were highest, traps were opened during the evening and night (from 18:00 to 8:00).

Avian predation was excluded by covering the experimental grids with nylon netting (6 cm mesh size) at a height of 1.5 m from the ground level. This allowed predatory mammals such as weasels, stoats, and red foxes to access freely. The control grids were accessible to all natural predators, including birds of prey. Each vole was individually marked by toe clipping when first captured. Upon each capture, sex, body mass (to the nearest 0.5 g) and reproductive condition of the vole were recorded before release at the point of capture.

### Ethical note

All experimental procedures described in this article were approved by the Third Local Ethics Committee for Animal Experimentation in Warsaw, Poland (WAW3/13/2004), and were conducted under licence from the Biebrza National Park (decision no. 20/O/200).

All methods were carried out in accordance with relevant guidelines and regulations.

All methods are reported in accordance with ARRIVE guidelines.

### Statistical analyses

To investigate whether preventing predation by raptors affected survival, we used individual capture histories to estimate apparent survival and its changes over the study period with a capture-mark-recapture (CMR) model. In addition, we analysed the effect of vole body mass on survival in the treatment and control plots, and finally, we looked at the effect of raptor removal on vole population dynamics. All analyses were performed using the R environment (version 4.4.1 ^59^;). Figures were prepared using R base graphics.

#### Estimation of survival and population size

We estimated monthly apparent survival using robust design models with Huggins conditional likelihood^60^. Within 5-day trapping sessions, populations are considered closed (no mortality or emigration is assumed), and survival was estimated between sessions. We applied an information-theoretic model averaging procedure by generating a set of candidate models, each including a subset of all considered parameters. In these models, survival (*S*) could be a function of treatment, location, time and an interaction of these variables, as well as age, cohort, sex and body mass of the individual, and the interaction of these variables with treatment; the effect of sex and body mass was additionally allowed to vary over time. Probability of capture (*p*) was modelled including the effects of location and sex, both interacting with time, and a linear effect of time within session (i.e. day of trapping session), possibly varying between sessions and location. Age and cohort were included as categorical variables. The effect of time (in primary periods, i.e. between trapping sessions) was included either as a categorical variable (i.e. session) or as a smooth spline function of time (B-spline based, with 3 or 4 degrees of freedom) to limit the number of parameters to be estimated. For the survival model, the categorical time variable interacting with location combined the last two sessions due to the parameter estimability problems in the last session. In the first session, there was no net set up (no treatment effect), hence all plots share the parameters for “control” group. All models considered included the effects of location and time; the survival model additionally included body mass and location-treatment interaction, and the capture probability model also included time-location interaction. The survival parameter (*S*) reported refers to a 30-day period, i.e. monthly rate. The parameters for first capture and recaptures were assumed to be equal (*p* = *c*), and temporary emigration was modelled as a uniformly random process (*γ*′ = *γ*″). We limited the number of model terms (including interactions) in each model to a maximum of 11; furthermore, we included only models in which all parameters were estimable and not at the boundary (i.e. not at 0 or 1, and with non-zero variance).

We assessed overdispersion in the models using parametric bootstrap^60^, in which the estimated parameters are used to simulate capture histories for each individual in the original sample, then the model is fitted to these simulated data, and the deviance is recorded. Goodness of fit was assessed as the proportion of deviances from the simulations exceeding the observed deviance. Overdispersion (*ĉ*) was calculated as the proportion of the observed deviance to the mean of the deviances from simulations. We present averaged model predictions (from models ranked highest by the small-sample Akaike Information Criterion, AIC_*c*_), with 95% confidence intervals. To obtain the expected values and confidence intervals of the model-averaged predictions, we used simulations from models’ *β* parameters, with number of replicates from each model proportional to the model weight, *ω*. This method was used to calculate all reported point estimates and their uncertainty, including derived parameters such as population size, its differences and relative survival. The *p*-values were calculated by taking twice the proportion of sample values falling beyond the distance between the sample value and the null value. For the prediction, we took the actual mean body mass and sex ratio of the captured individuals at the prediction point, e.g. session and/or location (rather than the overall mean). The CMR modelling was conducted with the program Mark^61^ version 10.1 (March 2023) through RMark interface (version 3.0.0 ^62^;).

#### Body mass estimation

Body mass was included in the CMR model as an individual covariate. Individual covariates need to be specified for each capture occasion, regardless of whether the individual was captured or not. Body mass changes dynamically throughout an individual’s lifetime, and while its dynamics are individual-specific, visual examination of the data revealed that they follow a limited number of temporal patterns. To approximate the body mass change over the entire study period for each individual in the data set, we used latent class linear mixed-effects models (LCME) to assign each individual to a pattern of body mass change. This assignment was based on the individual’s body mass at each capture occasion (trapping session) interacting with sex. We fitted models with 3 and 4 latent classes (i.e. body mass change patterns), with each model type replicated five times with different random starting values. For each model we derived a model weight from its Bayesian Information Criterion (BIC)^63^.

For each class and sex, we calculated the mean body mass of individuals in each session. Rather than assigning an individual to a single class by taking the highest class probability, we calculated the mean weighted by class probabilities, according to the formula:

1 $${\overline{m}}_{g,s,t}=\frac{{\sum }_{i\in {N}_{s,t}}{m}_{i,t}P({c}_{i}=g){m}_{i,t}P({c}_{i}=g)}{{\sum }_{i\in {N}_{s,t}}P({c}_{i}=g)},$$

where ${\overline{m}}_{g,s,t}$ is the average body mass in class *g*, per each sex *s* at occasion *t* for a set of individuals *i* that are of sex *s* and were caught at occasion *t*; *m*_*i,t*_ is the body mass of individual *i* at occasion *t* and *P(c*_*i*_ = *g)* is the probability of individual *i* belonging to class *g*. We calculated ${\overline{m}}_{g,s,t}$ for each model and then averaged the values considering models’ BIC weights. Since one model (with 4 classes) had a weight of almost 100%, equations presented here refer to single model estimates.

Next, to estimate body mass for each individual at all occasions, we adjusted ${\overline{m}}_{g,s,t}$ by adding the mean difference of their actual body mass to the mean at the occasions they were captured.

2 $${\widehat{m}}_{i,t}={\sum }_{g=1}^{k}({\overline{m}}_{g,{s}_{i},t}P({c}_{i}=g))+\frac{{\sum }_{t}({\overline{m}}_{g,{s}_{i},t}-{m}_{i,t}){I}_{i}(t)}{{\sum }_{t}{I}_{i}(t)},$$

The first part of Eq. 2 is the average of mean body masses over all classes *g*, for sex *s* and occasion *t,* weighted by the probabilities of individual membership in each of the latent classes. The second part is the mean difference between the respective mean body mass ${\overline{m}}_{g,s,t}$, and actual body mass, *m* of individual *i* at occasion *t*, where *I*_*i*_*(t)* is an indicator yielding 1 if individual *i* was caught at occasion *t*, or 0 otherwise.

The correlation between observed and estimated body mass used for the CMR model was *r*_*p*_ = 0.98 (Pearson’s correlation, *t* = 262, df = 2715, *p* ≪ 0.0001). The above method provided body mass estimates that deviated considerably less from the actual values than the prediction from model parameters. LCME model fitting was performed using R package ‘lcmm’ (version 2.1.0 ^64^).

## Supplementary Information

Below is the link to the electronic supplementary material.

Supplementary Material 1

## Data Availability

Data and code used in this study is available as supporting information on Zenodo at [10.5281/zenodo.16034731].
