# Supplementary material for: Avian predation has the strongest impact on vole survival during winter and spring in temperate grasslands
Source: Sci Rep. 2025 Dec 6;16:561. doi: 10.1038/s41598-025-30214-y (PMC12774968; doi:10.1038/s41598-025-30214-y)
Supplement: Supplementary file 1 — Supplementary Material 1 [file 41598_2025_30214_MOESM1_ESM.pdf]

Supplementary material

for „Avian predation has the strongest impact on vole survival during winter and spring in temperate grasslands”

Additional figures

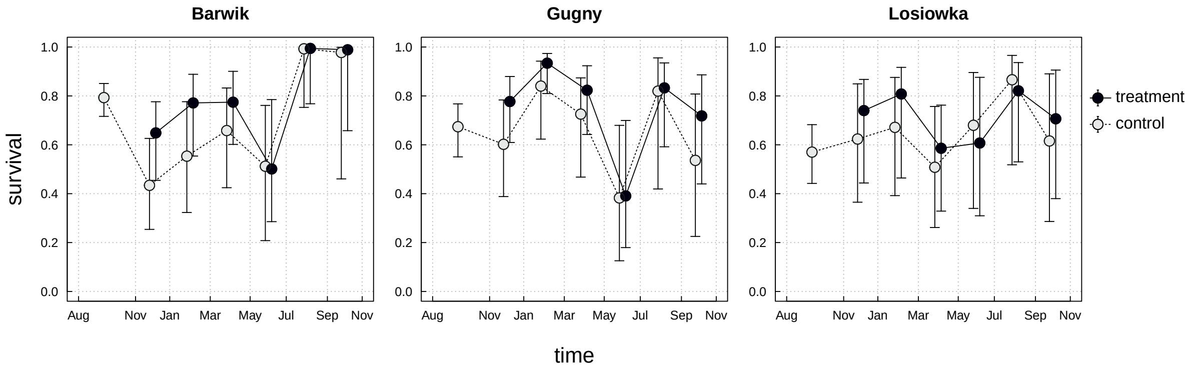

Figure S1. Survival of root voles at each site during the study, in treatment (no bird predation) and control plots, for intervals between sessions and scaled to a one-month period. Points show expected values, whiskers represent 95% confidence intervals. Estimates are averaged over a set of highest-ranked models (see see Table 1 in the main text). For better readability, consecutive points from the same plot are connected by a line, and points from the same session are slightly shifted apart along the horizontal axis.

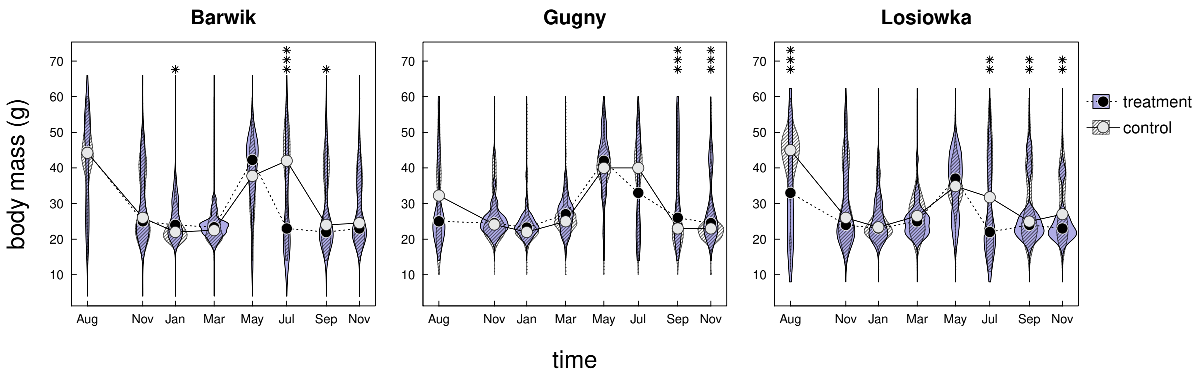

Figure S2. Body mass of voles captured in control and treatment plots, in each session and separately for each site. Violin graphs show the density distribution of body mass of individuals captured in each session, with dots indicating the median for control and treatment plots. or better readability, consecutive points from the same plot are connected by a line. Asterisks at top indicate significant difference (p ≤ 0.001, 0.01, 0.05 indicated by three, two and one symbols, respectively).

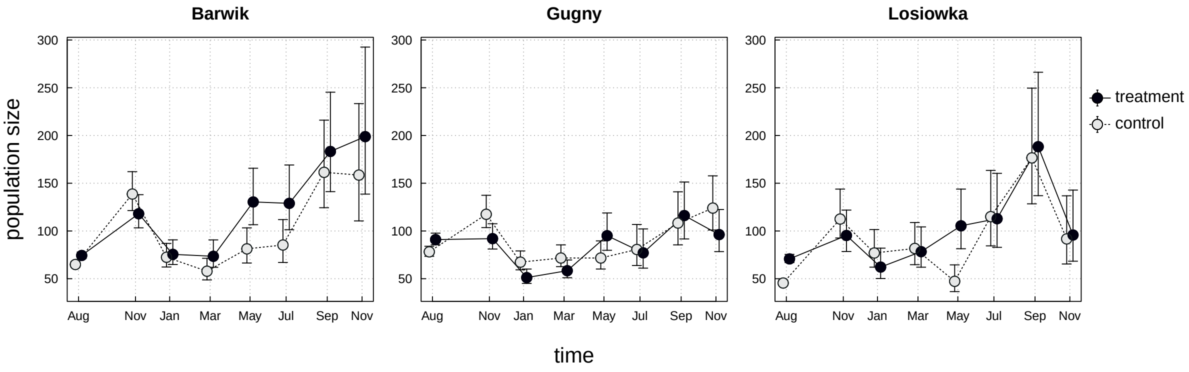

Figure S3. Population size of root vole at each site during the study in treatment and control plots. Points show expected values, whiskers represent 95% confidence intervals. Estimates are averaged over a set of highest-ranked models (see Table 1 in the main text). For readability, consecutive points from the same plot are connected by a line, and points from the same session are slightly shifted apart along the horizontal axis.

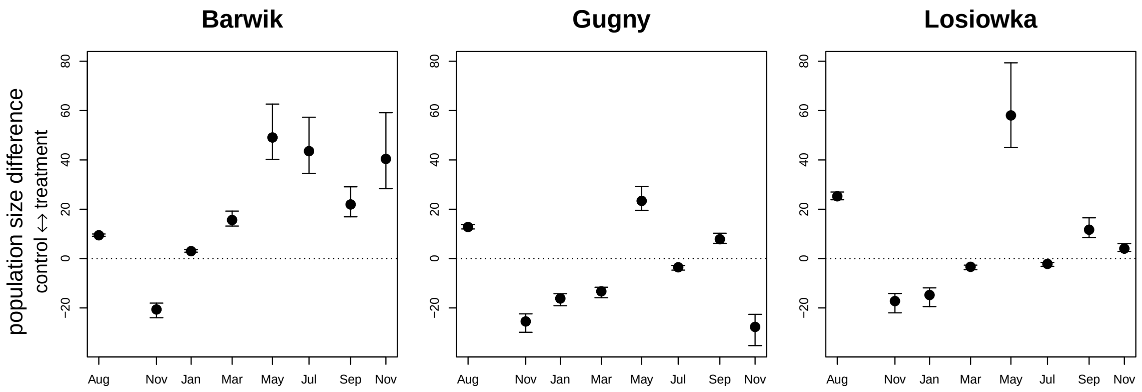

Figure S4. Difference in population sizes between control and treatment plots. Positive values reflect greater number of voles estimated in treatment plot. Points show expected values, whiskers represent 95% confidence intervals.

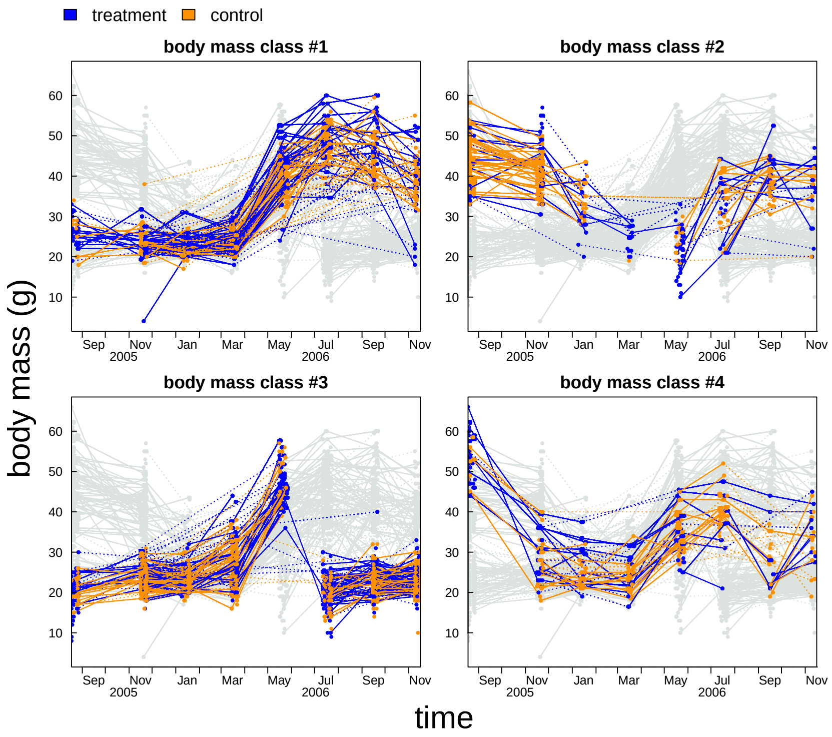

Figure S5. Changes in body mass of captured voles. The recorded body mass of individuals is shown as points connected by a line along the time axis. The colours indicate the individuals seen in the treatment and control plots. If an individual was captured in consecutive sessions, the points are connected by a solid line, whereas if the captures were discontinuous (the individual appeared in the preceding and subsequent sessions), the points are connected by a dashed line. Each panel highlights one group as determined by the latent class linear mixed model. To allow for comparison, the remaining groups are shown in the background in light gray.

Additional tables

Table S1. Number of voles caught in each plot in each trapping session.

Location	Type	Session date							
		18 Aug 2005	16 Nov 2005	9 Jan 2006	14 Mar 2006	16 May 2006	12 Jul 2006	15 Sep 2006	9 Nov 2006
**Barwik**	**treatment**	55	80	50	47	77	68	84	84
**Gugny**		64	65	38	44	65	44	59	59
**Losiowka**		56	61	38	47	58	53	80	47
**Barwik**	**control**	48	94	48	37	48	45	74	67
**Gugny**		55	83	50	54	49	46	55	76
**Losiowka**		36	72	47	49	26	54	75	45

Table S2. Number of voles known to be alive: in a given trapping session (MNA_t), known to have survived to the next session ($MNA_{t \rightarrow t+1}$), or known to have survived to the second subsequent session ($MNA_{t \rightarrow t+2}$); and respective recapture rates. Recapture rates were calculated as the ratio of individuals known to have survived to the (first or second) subsequent session to the current MNA.The numbers are broken down by location, plot type (treatment or control), and body mass class. The class membership of individuals was estimated using a linear mixed latent class model. The number of voles in each class was calculated based on the probability of membership of individuals to each class, hence the non-integer values. Highlighted are the winter recapture rates revealing large differences between treatment and control plots described in the Discussion.

time / time interval	class	treatment	MNA								recapture rate							
			Aug	Nov	Jan	Mar	May	Jul	Sep	Nov	Aug	Nov	Jan	Mar	May	Jul	Sep	Nov
current session (t)	1	control	16	59	55	56	65	65	46	31								
		treatment	41	66	63	62	78	58	51	34								
	2	control	76	60	18	10	21	24	33	21								
		treatment	31	41	17	14	38	28	24	22								
	3	control	27	96	73	63	28	51	113	110								
		treatment	67	83	64	58	62	98	147	116								
	4	control	20	39	31	34	37	33	40	25								
		treatment	36	35	33	33	41	17	31	18								
survived till subsequent session (t→t+1)	1	control	8	36	38	38	46	37	24		52%	62%	69%	69%	70%	57%	53%	
		treatment	21	52	53	42	46	40	26		50%	79%	84%	68%	58%	69%	52%	
	2	control	35	10	3	3	9	15	9		46%	**16%**	**16%**	32%	41%	62%	28%	
		treatment	13	10	9	6	10	18	14		41%	**25%**	**51%**	40%	26%	62%	56%	
	3	control	14	43	34	15	3	25	39		52%	44%	47%	24%	10%	48%	35%	
		treatment	23	53	42	31	9	57	70		34%	64%	66%	53%	15%	58%	48%	
	4	control	4	18	20	20	19	16	16		20%	46%	64%	60%	51%	50%	41%	
		treatment	8	26	24	19	13	10	12		24%	75%	74%	57%	32%	62%	39%	
survived till second subsequent session (t→t+2)	1	control	7	29	29	29	29	23			43%	49%	53%	52%	45%	35%		
		treatment	18	45	37	27	34	24			43%	67%	58%	43%	43%	42%		
	2	control	9	1	3	3	7	6			12%	**2%**	14%	31%	32%	24%		
		treatment	6	5	4	3	10	11			20%	**12%**	23%	21%	26%	40%		
	3	control	10	27	8	3	3	14			38%	28%	11%	5%	10%	27%		
		treatment	19	38	23	8	7	35			28%	46%	37%	13%	12%	36%		
	4	control	0	14	13	12	12	11			0%	35%	43%	36%	31%	33%		
		treatment	7	20	14	8	9	6			20%	57%	42%	23%	22%	35%		

Table S3. Number of captures (i.e. individuals seen for the first time) and re-captures (second or subsequent record) in each session, broken down into treatment and control plots, locations and as an overall total.

session	treatment		control		Barwik		Gugny		Losiowka		total	
	captures	recaptures	captures	recaptures	captures	recaptures	captures	recaptures	captures	recaptures	captures	recaptures
**18 Aug 2005**	175		139		103		119		92		314	
**16 Nov 2005**	160	46	193	56	130	44	108	40	115	18	353	102
**9 Jan 2006**	35	91	71	74	44	54	23	65	39	46	106	165
**14 Mar 2006**	39	99	68	72	36	48	23	75	48	48	107	171
**16 May 2006**	122	78	74	49	84	41	55	59	57	27	196	127
**12 Jul 2006**	123	42	98	47	75	38	61	29	85	22	221	89
**15 Sep 2006**	128	95	139	65	99	59	69	45	99	56	267	160
**9 Nov 2006**	68	122	99	89	103	0	119	0	92	0	167	211
**total**	850	573	881	452	626	380	539	367	566	278	1731	1025

Table S4. Number of captures in each session, brokend down by a cohort. In this context, "cohorts" are individuals first observed during each session, i.e. as it is used in the capture-mark-recapture model context.

session date	18 Aug 2005	16 Nov 2005	9 Jan 2006	14 Mar 2006	16 May 2006	12 Jul 2006	15 Sep 2006	9 Nov 2006
number of captures								
cohort 1	314	102	54	32	24	8	7	9
cohort 2		353	111	106	55	22	19	15
cohort 3			106	33	14	7	7	4
cohort 4				107	34	11	8	7
cohort 5					196	41	32	25
cohort 6						221	87	69
cohort 7							267	82
cohort 8								167

Table S5. Body mass of captured individuals in each session, and sex.

session	body mass [mean (s.d.)]		
	total	females	males
**August**	37.1 (13.0)	34.3 (11.8)	41.2 (13.7)
**November**	28.3 (8.7)	27.6 (8.0)	29.3 (9.4)
**January**	24.5 (4.7)	24.6 (4.9)	24.3 (4.5)
**March**	25.3 (4.4)	23.8 (3.7)	27.1 (4.4)
**May**	38.6 (8.8)	36.0 (8.1)	41.6 (8.5)
**July**	33.1 (13.1)	33.5 (11.9)	32.4 (15.0)
**September**	28.9 (10.5)	28.8 (9.9)	29.1 (11.3)
**November**	27.1 (8.1)	27.2 (7.8)	27.0 (8.5)

Table S6. Eetimates of population sizes at the beginning of the study (i.e. before netting), in control and treatment plots. Percentage difference refers to the mean population size of the pair of plots.

Location	median (95% CI)		difference of medians (n)	percentage difference
	control	treatment		
**Barwik**	64.7 (61.8 - 68.1)	74.2 (70.9 - 78.0)	9.4	14%
**Gugny**	78.3 (73.3 - 84.3)	91.1 (85.3 - 98.1)	12.8	15%
**Losiowka**	45.2 (42.8 - 48.4)	70.3 (66.6 - 75.3)	25.1	43%
**(total)**	64.8 (43.3 - 82.4)	74.5 (67.4 - 95.9)	9.7	14%
